# Supplementary material for: Preclinical Efficacy of a Capsid Virus-like Particle-Based Vaccine Targeting IL-1β for Treatment of Allergic Contact Dermatitis
Source: Vaccines (Basel). 2022 May 23;10(5):828. doi: 10.3390/vaccines10050828 (PMC9143278; doi:10.3390/vaccines10050828)
Supplement: Supplementary file 1 [file vaccines-10-00828-s001.zip › vaccines-1711157-supplementary.pdf]

Supplementary figures

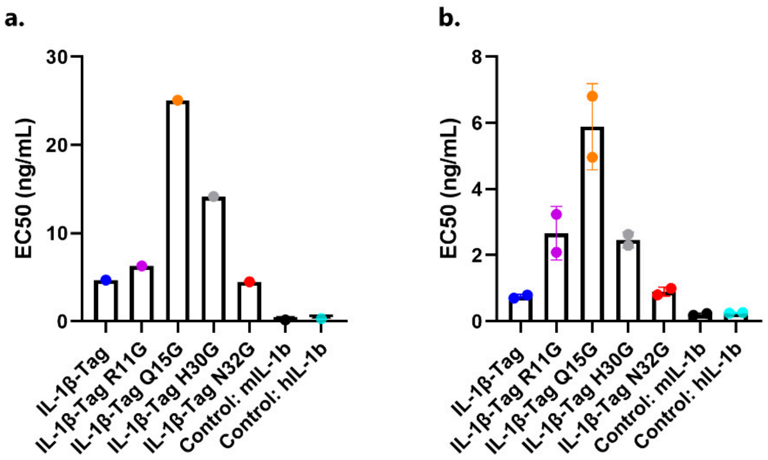

Figure S1 IL-1β biological activity EC_{50} values. Two technical replicates of the HEK293 cell-based IL-1β activity assay. Dilution curves were fitted using [Agonist] vs. response – variable slope (four parameters) equation. EC_{50} values were estimated from each fit.

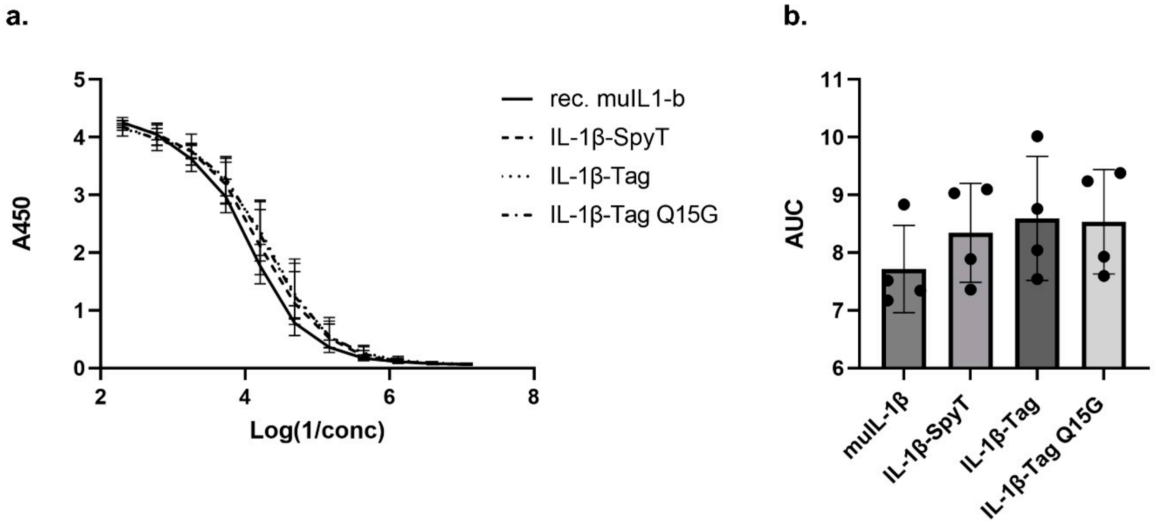

Figure S2 Test of IL-1β coat proteins against serum from mice vaccinated with cVLP:IL-1β WT The reactivity of serum from mice vaccinated with cVLP:IL-1β (7µg dose, W22) (n=4) was tested against recombinant muIL-1β, IL-1β-SpyT, IL-1β-Tag or IL-1β-Tag Q15G. **a.** Total IgG anti-IL-1β dilution curve with geometric mean ± geometric SD. **b.** Anti-IL-1β total IgG titers depicted as AUC, with mean ± SD. Statistical analysis was performed on log-transformed AUC values using one-way ANOVA, Tukeys multiple comparisons test (adjusted p-value < 0.05 was accepted as significant).

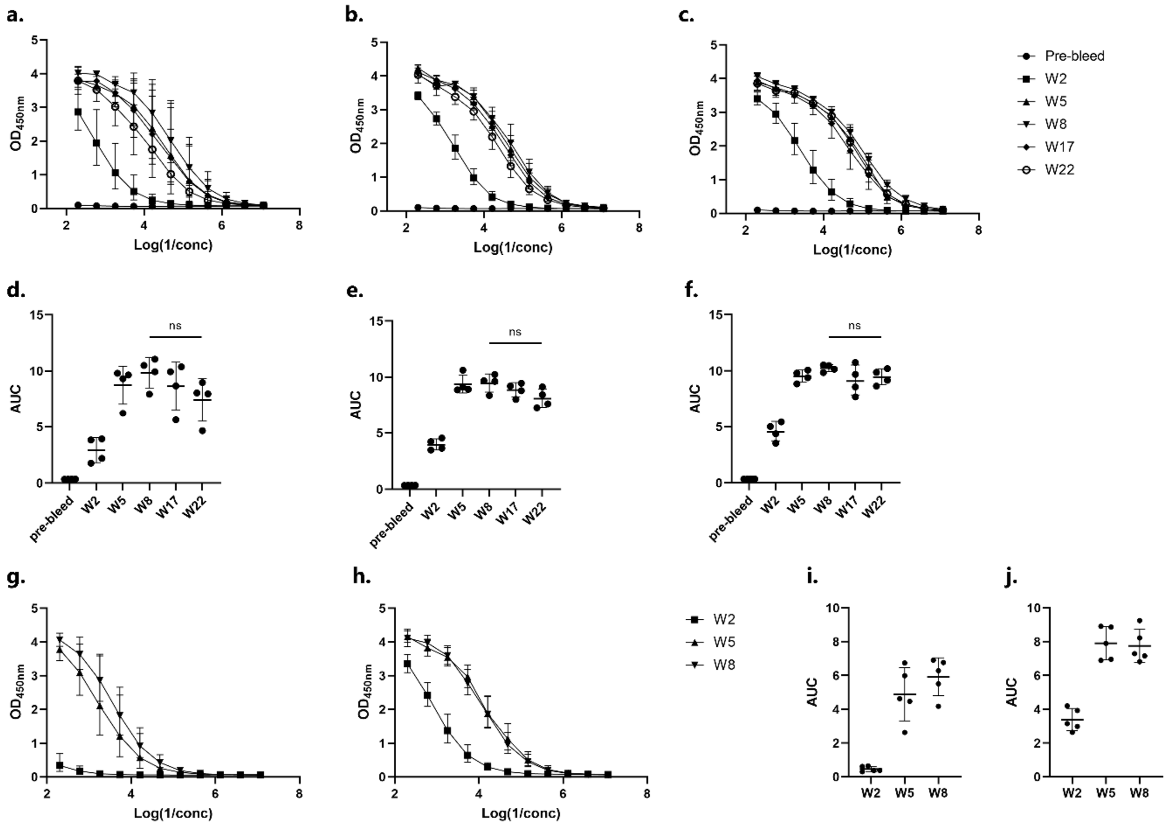

Figure S3 Total IgG titers measured in serum from mice vaccinated with cVLP:IL-1β vaccines. Serum samples were obtained from female C57BL/6 mice pre vaccination (pre-bleed) and two weeks after prime (W2), boost (W5) and second boost (W8), and additional blood samples at W17 and W22 for mice vaccinated with cVLP:IL-1β **a-c.** Total IgG anti-IL-1β dilution curve with geometric mean ± geometric SD for mice vaccinated with cVLP:IL-1β dose of 2µg IL-1β **(a)**, dose of 7µg IL-1β **(b)** or dose of 10µg IL-1β **(c)**. **d-f.** Total IgG anti-IL-1β titers depicted as AUC with mean ± SD from mice vaccinated with cVLP:IL-1β dose of 2µg IL-1β **(d)**, dose of 7µg IL-1β **(e)** or dose of 10µg IL-1β **(f)**. **g.** Total IgG anti-IL-1β dilution curve with geometric mean ± geometric SD for mice vaccinated with cVLP:IL-1β. **h.** Total IgG anti-IL-1β dilution curve with geometric mean ± geometric SD for mice vaccinated with cVLP:IL-1β Q15G. **i.** Total IgG anti-IL-1β titers depicted as AUC with mean ± SD from mice vaccinated with cVLP:IL-1β **j.** Total IgG anti-IL-1β titers depicted as AUC with mean ± SD from mice vaccinated with cVLP:IL-1β Q15G.

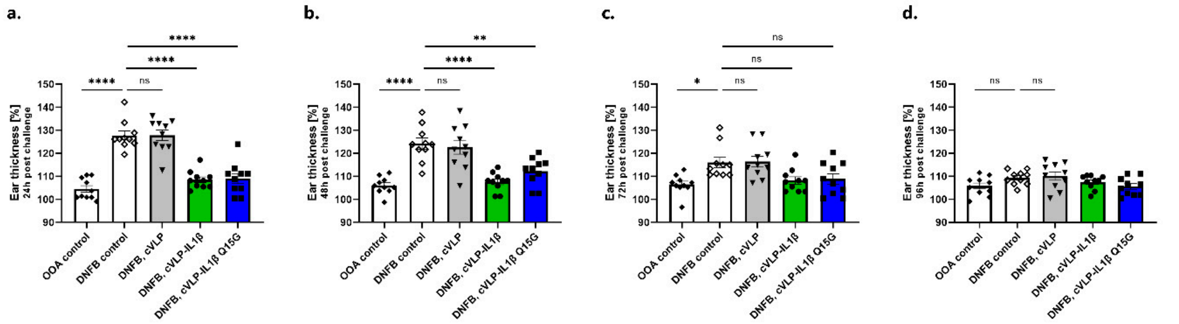

Figure S4 Individual measurements of ear thickness post challenge of mice. Ear thickness measured **a.** 24h, **b.** 48h, **c.** 72h and **d.** 96h post DNFB challenge, depicted as percentage (%) with mean ± SEM. Graphs show data from two technical replicates (n=5). Statistical analysis was performed on log-transformed values using one-way ANOVA, Tukeys multiple comparisons test (adjusted p-value < 0.05 was accepted as significant).

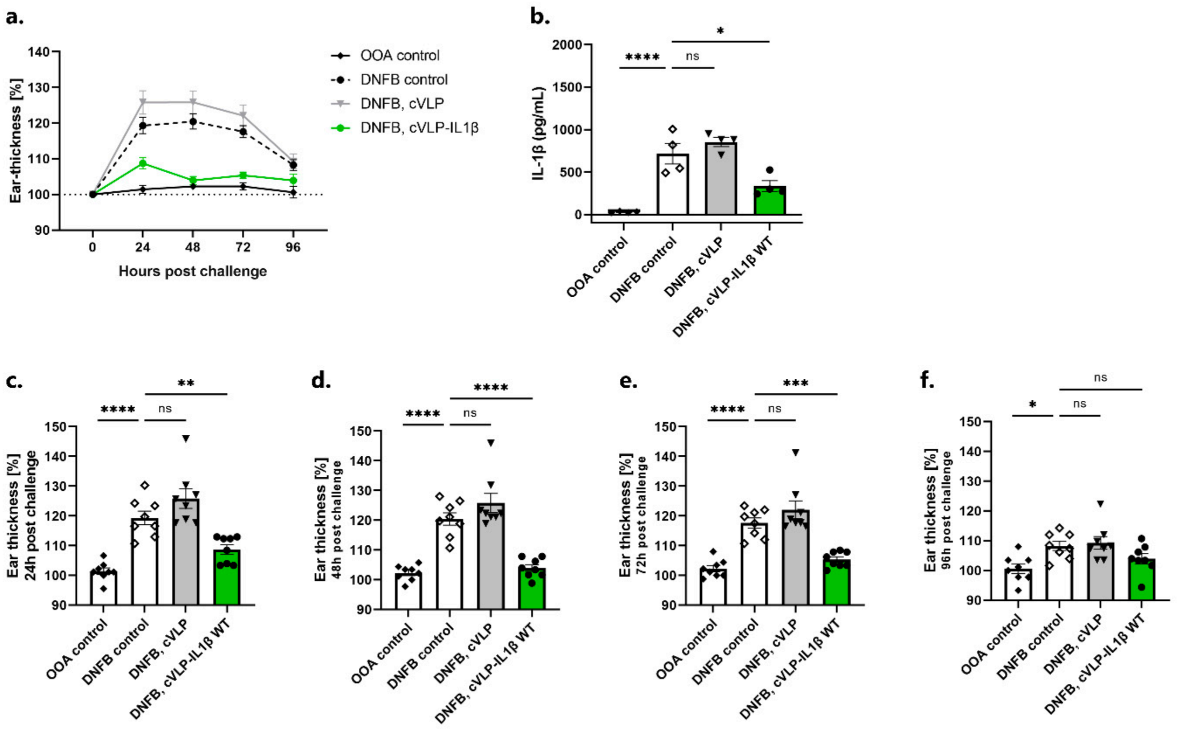

Figure S5 Challenge study for mice vaccinated with another cVLP:IL-1β vaccine batch. **a.** Ear thickness measured on groups of mice (n=8) 24h, 48h, 72h and 96h post challenge, depicted as percentage (%) with mean ± SEM. **b.** IL-1β levels with mean ± SEM measured in ears from groups of mice (n=4) sensitized and challenged with 1) OOA, 2) 0.15% DNFB, 3) 0.15% DNFB and vaccinated with unconjugated cVLP and 4) 0.15% DNFB and vaccinated with cVLP:IL-1β WT. **c-f.** Ear thickness measured 24h **(c)**, 48h **(d)**, 72h **(e)** and 96h **(f)** post DNFB challenge, depicted as percentage (%) with mean ± SEM. Statistical analysis was performed on log-transformed values using one-way ANOVA, Tukeys multiple comparisons test (adjusted p-value < 0.05 was accepted as significant).

3’ **R11G** primer	TTT CCA TGG GCC ATC ACC ATC ATC ATC ATG AAA ATC TGT ATT TTC AGG GTG TTC CGA TTC GTC AGC TGC ATT ATC GTC TG**G GC**G ATG AAC AGC AGA AAA GCC TGG
5’ PCR1 **Q15G** primer	CCA GGC TTT TGC CCT GTT CAT CAC GCA GAC GAT AAT GCA GC
3’ PCR2 **Q15G** primer	ATG AAC AG**G GC**A AAA GCC TGG TTC TGA GCG ATC CGT ATG AAC T
5’ PCR1 **H30G** primer	CCA TTC AGG CCC AGT GCT TTC AGT TCA TAC GGA TCG CTC AG
3’ PCR2 **H30G** primer	AAA GCA CTG **GGC** CTG AAT GGT CAG AAT ATT AAC CAG CAG GTC A
5’ PCR1 **N32G** primer	TAT TCT GAC CGC CCA GAT GCA GTG CTT TCA GTT CAT ACG GAT C
3’ PCR2 **N32G** primer	GCA TCT G**GG C**GG TCA GAA TAT TAA CCA GCA GGT CAT TTT CAG C

Table S1 Primers for introducing mutations into the muIL-1β gene sequence.
